# Dispersal Polymorphism and the Speed of Biological Invasions

**DOI:** 10.1371/journal.pone.0040496

**Published:** 2012-07-20

**Authors:** Elizabeth C. Elliott, Stephen J. Cornell

**Affiliations:** Institute of Integrative and Comparative Biology, University of Leeds, Leeds, United Kingdom; Centro de Investigación y de Estudios Avanzados, Mexico

## Abstract

The speed at which biological range expansions occur has important consequences for the conservation management of species experiencing climate change and for invasion by exotic organisms. Rates of dispersal and population growth are known to affect the speed of invasion, but little is known about the effect of having a community of dispersal phenotypes on the rate of range expansion. We use reaction-diffusion equations to model the invasion of a species with two dispersal phenotypes into a previously unoccupied landscape. These phenotypes differ in both their dispersal rate and population growth rate. We find that the presence of both phenotypes can result in faster range expansions than if only a single phenotype were present in the landscape. For biologically realistic parameters, the invasion can occur up to twice as fast as a result of this polymorphism. This has implications for predicting the speed of biological invasions, suggesting that speeds cannot just be predicted from looking at a single phenotype and that the full community of phenotypes needs to be taken into consideration.

## Introduction

There is evidence that species are expanding their range as a result of climate change and due to accidental or deliberate introductions of exotic organisms [1]–[4]. The speed at which a species is able to expand its range has important implications for conservation management. Whether a species can shift its range at the same rate as the climate shifts, or whether (and by how much) it lags behind, will be important in determining how likely a species is to survive a period of climate change [5], [6]. The rate of spread of exotic species as a result of introductions can also be important especially if these species become pests [7].

The speed of a species invasion depends upon its dispersal ability and population growth rate, which are affected by a number of demographic and environmental parameters. Many theoretical models have investigated species' invasion speeds under different conditions, beginning with the work of Fisher [8]. Developments since Fisher have found that many factors including Allee effects, timing of reproduction and dispersal in the life cycle and environmental heterogeneity can influence the speed of invasion (reviewed in [9]). Adaptation to local conditions has also been found to influence the rate of spread. García-Ramos and Rodríguez found that in a spatially heterogeneous environment the rate of local adaptation can be the key limiting factor to spread, with faster range expansions occurring when the environmental gradient is shallower [10].

During invasions there is a selection pressure for increased dispersal. The effect that the evolution of dispersal rate has on the speed of invasion has been investigated using an individual-based model (IBM) by Travis and Dytham [11]. They found that if dispersal evolves during an invasion then there is a faster rate of spread, with the rate that evolves determined by the cost of dispersal. Other studies have also revealed that during range expansions there is evolution towards increased dispersal resulting in faster rates of spread. The extent to which increased dispersal evolves can depend on different factors, with increased levels of evolution if there are no competitors present [12], if Allee effects are absent [11] and if there is a greater quantity of habitat available [13]. It has also been shown that the rate at which species expand their range can be affected by the way that dispersal is modelled. Faster range expansions occur when density-dependent strategies are allowed to evolve [14], when a dispersal kernel rather than just an emigration rate is evolving [15] and when there is temporal variability in dispersal [16].

There is also increasing empirical evidence from species that are expanding their range, of evolutionary adaptations related to dispersal ability in individuals in more recently colonised areas (review see [17]). For example, more recently colonised sites of the speckled wood butterfly, *Pararge aegeria*, contain populations with larger adults, greater thorax mass and broader thorax shape [13], [18], [19]. All of these traits are related to flight ability and so mean that these individuals may be more dispersive and hence may invade faster.

There is evidence, then, that individual dispersal phenotypes can evolve during range expansion, and that the speed of range expansion itself can also evolve. One study has shown that during a range expansion selection favors individual behavior that increases the rate of expansion for the population, rather than selecting for behavior that minimizes disperser mortality [20]. However, very little is known about how the rate of range expansion itself is related to the range of phenotypes that are present in the population. Classical models for computing invasion speeds (reviewed in [9]) consider a single phenotype, and it is not clear whether the speed of range expansion of a polymorphic population should be that of one particular phenotype (say, the most dispersive one), an average over phenotypes, or something else entirely. To this end, we study a model for invasion by a species with dispersal polymorphism, which is simple enough for the the invasion speeds to be computed explicitly. Our model is spatially-explicit, with mutation between two morphs which differ in their dispersal and demographic parameters and interact via Lotka-Volterra dynamics.

## Methods

We use a spatially explicit general Lotka-Volterra model which assumes that individuals disperse and reproduce randomly during the lifetime of the individual. We are also assuming that time is continuous and that the landscape is spatially and temporally homogenous. In this model there are the following two phenotypes that differ in their dispersal ability:

- an establisher morph 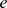 that after establishment has a higher growth rate but is a poorer disperser; and
- a disperser morph 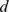 that has a lower growth rate after establishment but is a better disperser

This model describes the spatio-temporal dynamics for the population density of each of these morphs. Population density of the species is denoted by 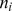 with subscript 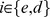 representing density of each morph. Morph 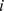 has dispersal rate 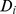 and growth rate 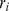. Density dependence follows Lotka-Volterra dynamics, with parameters 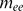 and 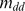 representing competition between individuals of the same morph, and 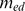 and 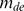 representing competition between the different morphs. Mutation at birth leads to a fraction of the offspring of one individual being of the other morph, which occurs at per-capita rates 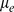 and 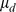 respectively (note that 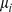 is the product of the per-capita birth rate and the mutation probability per generation). Thus the equations are given by:
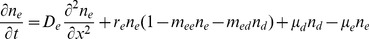
(1)

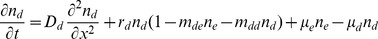
(2)


The first term on the right hand side of each equation describes the random dispersal of each morph. The second term describes the population growth of each morph where we have assumed that births are density independent and mortality is density dependent. The third and fourth term describe the mutation of morphs into each other i.e. the rate of production of offspring by either phenotype that is of the other type. A more detailed derivation of the model from explicit demographic processes is described in Appendix S1. We are interested in how the invasion speed depends on the model parameters.

We are primarily interested in the case where there is a cost to being a better disperser, so in the following we will implicitly assume 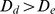 and 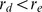. This implies that the disperser morph 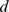 is better at dispersing but pays a cost by having a lower growth rate, and that 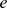 the establisher morph is a poorer disperser but has a higher growth rate. We have also analysed the case where one morph is both a better disperser and establisher; we do not discuss this case further as we find, unsuprisingly, that this superior morph always dominates.

We analysed this model using both analytical techniques and numerical simulations, studying the case where half of space is initially occupied at a stable equilibrium and the other half is completely empty. We first studied the invasion by each morph when present in the landscape on its own, without mutation into the other morph. We then studied the case where both morphs coexist in the landscape. We are interested in the biologically relevant case where the mutation rate is small, in which case closed-form analytical solutions were found.

Analytical results for the underlying partial differential equations were obtained by first finding the equilibrium population density of each morph and then using the method of front propagation to calculate the invasion speed [21]. The equations were then solved numerically by approximating the spatial derivatives by finite differences, so that the partial differential equations become a set of coupled ordinary equations, and then carrying out simulations in R [22] using the deSolve function [23]. These simulations produced a travelling wave at the invasion front which rapidly approached a constant speed as the invasion progressed. The invasion speed was estimated by calculating the distance that the density profiles at different times need to be displaced in order to lie on top of each other.

## Results

### Calculation of Invasion Speed

We first considered the case where each morph 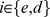 is present in the landscape on its own. In this case the system of equations (1) and (2) reduces to 
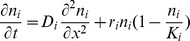
 where 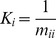 is the carrying capacity of morph 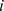. We looked for travelling wave solutions and found that the invasion speed, 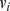, is determined by the morph's growth rate and dispersal ability: 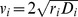. The invasion is faster when either the growth rate or dispersal ability is increased, as was originally found by Fisher [8].

We then investigated the case where both morphs were present in the landscape and there is mutation between them. In this case we used the front propagation method of van Saarloos [21] to calculate the invasion speed. The general system of spatially uniform equations has two equilibria: an unstable extinction state where 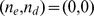, and a stable coexistence state which we will denote by 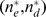. The front propogation method involves linearising the equations about the unstable steady state, which gives:
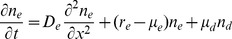
(3)

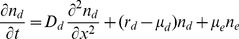
(4)


Note that these equations only depend upon the morphs' population growth rates, dispersal abilities and mutation rates, and are independent of the competition coefficients 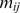. Here we are assuming that the speed we calculate using the linear system (3) and (4) also applies to the nonlinear system (1) and (2). This linear speed is known to be a lower bound of the invasion speed but it is not always exact [24]. In the numerical simulations section we will therefore check that the results we obtain based on this linear conjecture are valid.

Using these linearised equations, and following van Saarloos [21], we substitute 
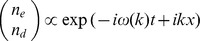
 where 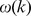 is the dispersion relation of Fourier modes of the linearised equations (3), (4) and 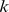 is the spatial wavenumber. This gives the equations:
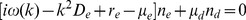
(5)

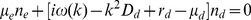
(6)


This leads to an eigenvalue problem, with solutions
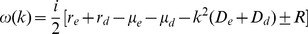
(7)where 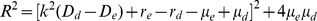. This implies



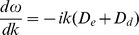


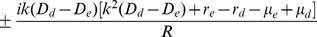



We then calculate the wave speed by finding 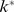, where 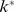 is the linear spreading point [21], such that
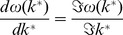
(8)where 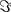 denotes the imaginary part. These equations represent a biological invasion so 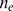 and 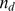 cannot be negative, so we can deduce that 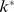 is purely imaginary. We can then assume 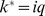 with 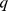 real, and substituting into (8) we get



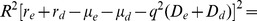


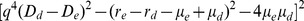
(9)


The realised wavespeed is obtained by finding the real solution 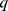 to Eqn. (9) that corresponds to the largest speed 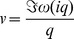 [21]. Eqn. (9) is a quartic equation in 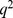, which can readily be solved numerically, though general analytical results are somewhat laborious to obtain. Simple analytical results can, however, be obtained in the biologically interesting limit of weak mutation between the morphs. Taking the limit 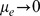 and 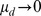 in Eqn. (9) we obtain
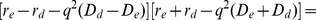


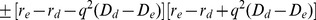
(10)


This gives three values for 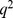: 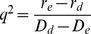, 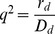 and 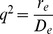, which after substitution into (8) gives three possible values for the limiting wavespeed:
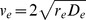
(11)

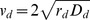
(12)

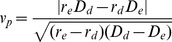
(13)


Note that 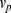 is only real if 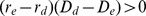, so this speed clearly does not occur if one morph is both a better disperser and a better establisher. For a finite but small mutation rate, we would expect the invasion speed to equal one of the above values plus a small correction proportional to some power of the mutation rate. 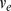 is the speed at which the establisher would invade in isolation, 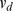 is the invasion speed of the disperser, and 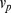 is a third wavespeed that is dependent on both morphs' establishment and dispersal abilities.

It is straightforward to show that 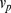 (provided it is real) is larger than either 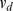 or 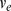, but we have not yet shown that this speed corresponds to an appropriate solution to the PDEs in the limit where the mutation rate is small but finite, for which 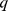 must be real. We assume that the mutation rates are small, but not necessarily the same, so we substitute 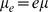, 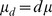, where *e* and 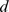 are positive. We substitute 
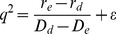
 into (9), where 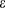 is small when the mutation rate is small. When we take the limit 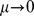, we find 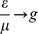, where
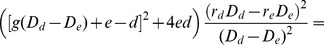


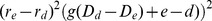


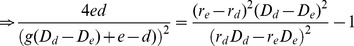



Since 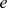 and 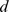 are both positive, 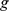 will be real if and only if
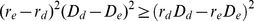



**Figure 1:**
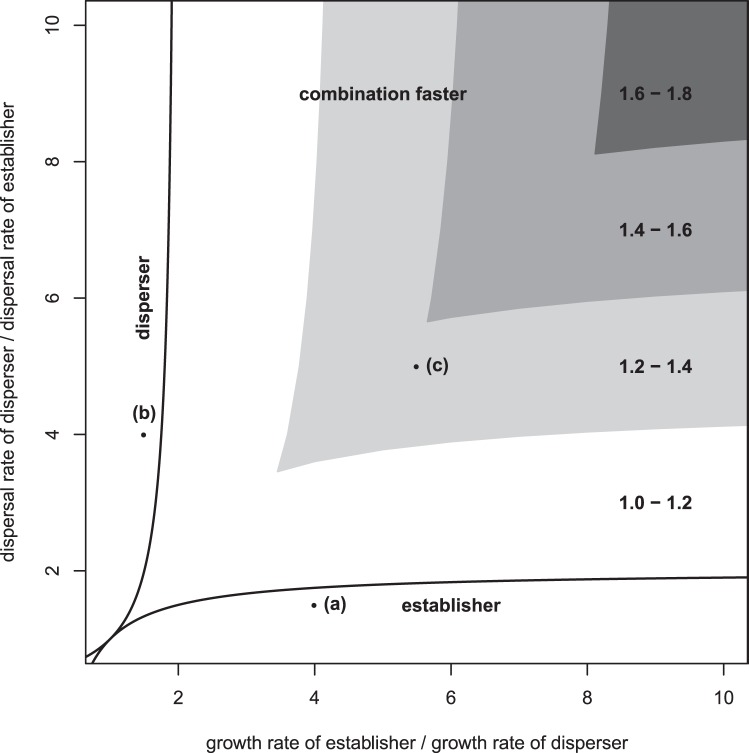
Parameter regions where each invasion speed occurs. The area between each of the curves (given by (14) and (15)) and the axes is where the polymorphic invasion occurs at approximately the same monomorphic speed as one of the phenotypes. The area above the curves is where the polymorphic invasion occurs faster than either monomorphic invasion, with the shading from white to grey representing the extent to which the polymorphic invasion is faster. (a), (b) and (c) show the parameters used in Fig. 3. The area where the ratio of the net growth rates and dispersal rates is less than one is not shown in this figure because that region of parameter space violates our assumption of net growth rate of establisher 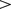 net growth rate of disperser and dispersal rate of disperser 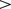 dispersal rate of establisher. If there were a tradoff in only one of the traits, for example, if both morphs have the same net growth rate but different dispersal rates then the ratio of growth rates would be 1 and so we can see that the invasion would follow the speed of the disperser. Similarly if the morphs only differed in their net growth rate then we can see that the invasion would follow the speed of the establisher.

This condition is satisfied, and therefore the invasion will proceed at speed 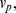 in the positive quadrant of 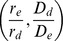 space that is bounded by the curves
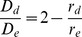
(14)and



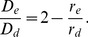
(15)Below the curve defined by (14), the invasion proceeds at the speed of the monomorphic establisher 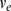, whereas the invasion will proceed at the monomorphic disperser speed 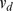 when parameters are above curve (15) in 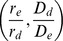 space. The parameter regions where each of these speeds occur are illustrated in Fig. 1.

The faster invasion speed occurs when the difference of both traits between morphs is roughly greater than a factor of two, and for the parameter regions shown can be up to twice as fast as the single phenotypes invasion speeds (Fig. 1). Polymorphic invasion speeds could be more than twice as fast as either monomorphic speed if the morph's parameters differ by a factor of more than ten, but this is unlikely to be the case for real species.

### Numerical Simulations

Numerical simulations were carried out to determine whether the analytical results using the linear conjecture do indeed give the true wave speeds for the nonlinear system of equations (1) and (2). The simulations were carried out by approximating the spatial derivatives by finite differences and then carrying out simulations in R [22]. We first studied the case where both morphs have the same carrying capacity and interact neutrally (
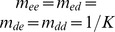
).

**Figure 2:**
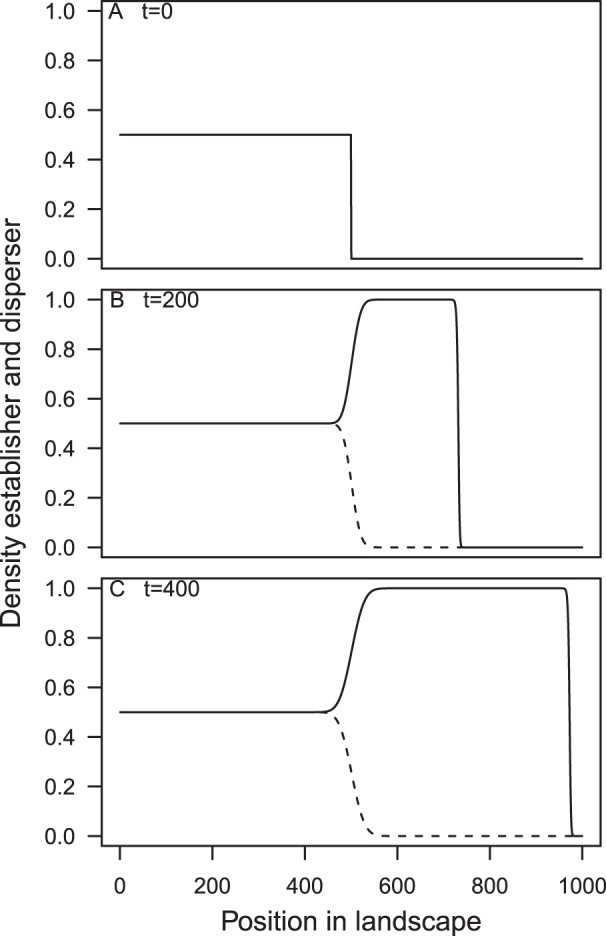
Invasion profile when there is no mutation between phenotypes. At the start of the simulation half of the landscape was filled with both morphs each at half of the carrying capacity and the other half of the landscape was unoccupied. This is an example of the case where the establisher morph (solid line) has the fastest single invasion speed and so this phenotype invades through the landscape and the disperser morph (dotted line) remains in its initial location. The parameter values used for this simulation were 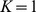, 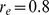, 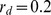, 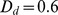, 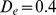.

Initially we carried out simulations with no mutation between morphs. In this case both coexist neutrally at equilibrium, however, during an invasion whichever morph has the faster invasion speed in isolation will invade the empty landscape and reach its carrying capacity (Fig. 2). The interface between coexistence of phenotypes and dominance of the faster morph moves very slowly (Fig. 2). We do not observe a wave travelling at speed 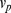 in this case because the two morphs are not invading the landscape at the same speed.

**Figure 3:**
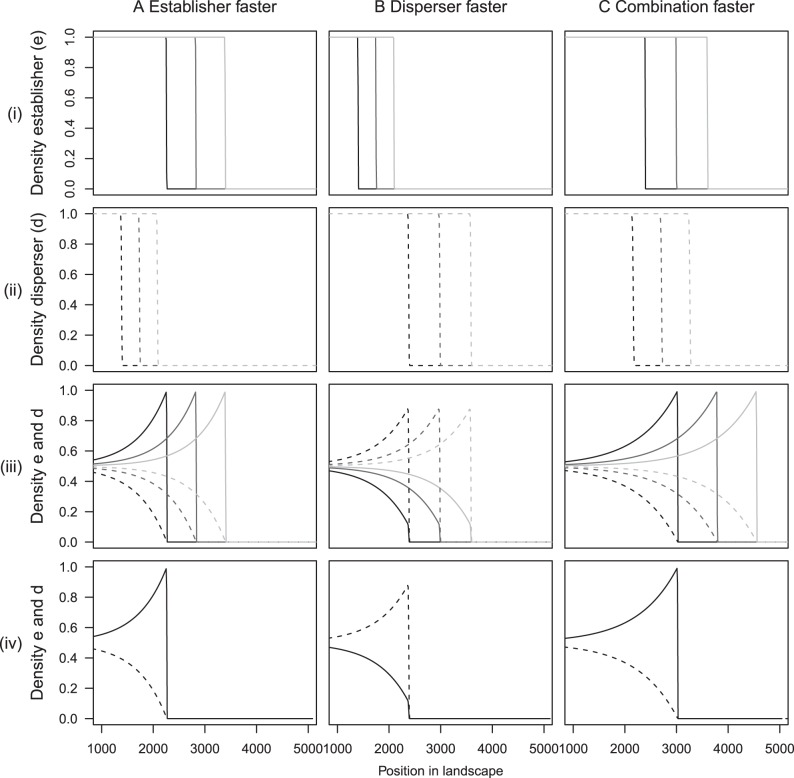
Invasion profiles of the two morphs. These show the establisher morph (solid line) and disperser morph (dashed line) when present in the landscape on their own (rows (i) and (ii)) and when mutation allows both to be present (row (iii)). Row (iv) shows the same data as row (iii), but the invasion waves are shifted by (speed) times (time) to illustrate that the wave maintains its shape as it travels. The simulations were initiated with the first 100 cells occupied by each phenotype at its equilibrium population density and the remaining cells unoccupied. The simulations were run on a lattice consisting of 8000 cells, using a space increment of 0.1. For all graphs each line represents the density profiles at a different time point, shown by the different shades of grey, with each time point 500 units apart. In column (A) the polymorphic invasion speed is the same as the monomorphic establisher speed; in column (B) the polymorphic invasion speed is the same as the monomorphic disperser speed, and in (C) the polymorphic invasion speed is faster than either monomorphic invasion. For all simulations 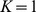, 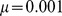, and in (a) 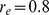, 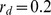, 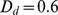, 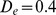; (b) 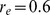, 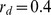, 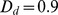, 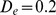; (c) 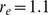, 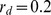, 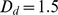, 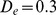.

We found that the addition of a small amount of mutation between phenotypes allows both morphs to coexist during an invasion. Fig. 3 illustrates examples where invasions occur at the three different speeds that were found analytically, for neutral interactions and equal small mutation rates between morphs. These three invasion speeds occur as a result of differences in the dispersal and establishment abilities of the two phenotypes. When the dispersal abilities of the disperser and establisher are similar but the population growth rate of the establisher is much higher than that of the disperser the invasion occurs at the speed of the establisher (Fig. 3A). When the population growth rates of each strain are similar but the dispersal rate of the disperser is much higher than the dispersal rate of the establisher the invasion occurs at the speed of the disperser (Fig. 3B). However, when there is a big difference between the two phenotypes in terms of both the dispersal and establishment abilities, the invasion occurs faster than either single morph (Fig. 3C).

**Figure 4:**
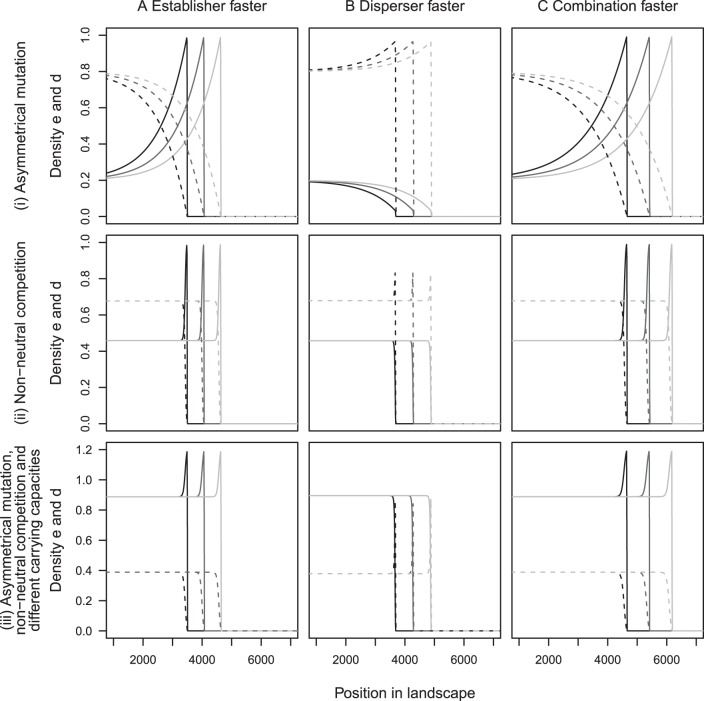
Invasion profiles with different parameter values. These show that invasion speeds are the same when (i) morphs have asymmetrical mutation rates, (ii) there is non-neutral competition and (iii) morphs have asymmetrical mutation, non-neutral competition and different carrying capacities. The simulations were initiated with the first 100 cells occupied by each phenotype at its equilibrium population density and the remaining cells unoccupied. The simulations were run on a lattice consisting of 8000 cells, using a space increment of 0.1. For all graphs each line represents the density profiles at a different time point, shown by the different shades of grey, with each time point 500 units apart. In column (A) the polymorphic invasion speed is the same as the monomorphic establisher speed; in column (B) the polymorphic invasion speed is the same as the monomorphic disperser speed, and in (C) the polymorphic invasion speed is faster than either monomorphic invasion. For all the simulations the parameters used are the same as in Fig. 3 apart from in (i) have that 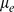 = 0.001 and 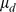 = 0.00025, in (ii) 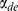 = 0.8, 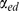 = 0.7, where 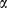 is the competition coefficient, and in (iii) have both the parameters used in (i) and (ii) and additionally 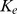 = 1.2 and 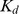 = 1.

The analysis predicts that the invasion speed is independent of the carrying capacities and mutation rates, provided the mutation rate is small. To check this, we also carried out simulations with different mutation rates between morphs (Fig. 4i), non-neutral interactions (Fig. 4ii), and non-neutral interactions with different mutation rates and different carrying capacities (Fig. 4iii). The case where the morphs differ only in their carrying capacity is not shown, because in that case the two morphs do not coexist in the absence of mutation. As predicted, we found that none of these parameters has an effect on the invasion speed, though they could change the shape of the invasion profile. Only the morphs' population growth rates and dispersal abilities were found to affect the species' invasion speed.

**Figure 5:**
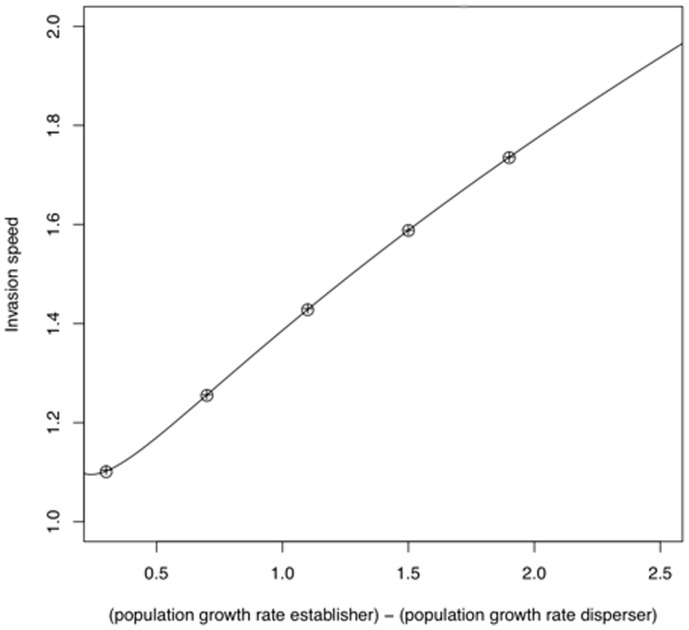
Comparison of analytical and numerical predictions of the invasion speed. This is an example of the case when polymorphism results in faster invasions than either single morph. The curve represents the analytical predictions of the invasion speed in the limit 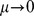 given by Eqn. (13). The crosses represent numerical predictions calculated numerically using Eqn. (10), and the circles the numerically integrated predictions, when 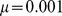 using a space increment of 0.1. Parameter values used were 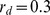, 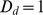, 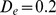 and 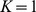. For the analytical prediction 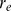 was varied and for the numerical simulations the values of 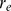 used were 0.6, 1, 1.4, 1.8 and 2.2.

The invasion speeds found in numerical simulations are compared to the analytical predictions in Fig. 5. The formulae for the invasion speeds (11–13) apply strictly in the limit 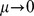, so to confirm that these formulae can be used for finite 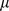 we have also included numerical solutions to Eqn. (10) for 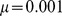. We find excellent agreement between the invasion speed from numerical simulations, analytical predictions for 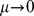, and numerical predictions from the wave propagation method for finite 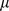.

## Discussion

We have investigated the effect of the presence of two dispersal phenotypes on a species' invasion speed. We found that, if the morphs differ in both their dispersal ability and growth rate, then the invasion speed can be faster than the speed of either morph on its own. This effect becomes significant when both traits differ by a factor of two or more, and for reasonable parameter values the combined invasion speed will be up to twice as fast as either monomorphic speed. Surprisingly, this effect persists when the mutation rate between the morphs is vanishingly small, even though this should mean that each morph has a minuscule effect on the other morph in the leading edge of the invasion front. We have shown that this effect is robust to the other parameters in the system, such as non-neutral interactions and differences in mutation rates.

Mathematically, there is no reason to expect that the invasion speed of a community of phenotypes should follow the speed of any particular phenotype. For example, the invasion speed obtained by solving Eqn. (9) does not equal 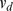 or 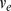 when the mutation rate is finite. Weinberger *et al.* [25] have reported an example where a polymorphic population invades faster than any of its constituent phenotypes in isolation. They studied a model of a two-allele, one-locus diploid species, and found that the presence of both homozygotes and heterozygotes results in the homozygotes spreading at a faster speed than they do in the absence of the heterozygotes but which is also faster than the invasion speed of the heterozygotes [25]. However, in Weinberger's model there are strong `cooperative' effects between the phenotypes, since a significant fraction of offspring of heterozygotes will be homozygotes. Since our anomalous speeds persist when the mutation between morphs is vanishingly small, we have shown that faster speeds are also observed in competing systems where there is effectively no cooperation.

It is technically difficult to prove that the linear wave speed correctly describes the speed of travelling waves in reaction-diffusion systems, and since our model is not an example of the cooperative systems studied in [24], [25] there is no proof that Eqn. (13) is the correct invasion speed for the dimorphic system. Nevertheless, we can prove that the dimorphic wave speed is indeed higher than the speed of either monomorphic system. This follows because the linear wave speed is, in general, a lower bound to the true wave speed [24]; meanwhile, the monomorphic case reduces to the Fisher wave equation – for which the linear speed is known to be the true wave speed. Therefore, the dimorphic system invades at a speed faster than Eqn. (13), which is itself faster than either Eqn. (11) or (12).

Our model is quite generic, and we would expect this phenomenon to occur in a very wide class of systems because the method for predicting the wave speed depends solely on the linearised behaviour of the partial differential equations close to the unstable equilibrium [21]. Clearly, there will be many different systems of reaction-diffusion equations which have the same linearised form at low density. For instance, if density dependence acts on birth then the mutation term in Eqns. (1–2) would be density dependent, but the linearised form of the equations would be the same as when density dependence acts on mortality. Reaction-diffusion equations of this type can also be used to model spatially explicit metapopulation dynamics, since colonisation-extinction dynamics are mathematically equivalent to birth-death-dispersal processes [26], [27]. In that case, density dependence acts on the colonisation process through the availability of unoccupied patches, which means that: (i) interactions between morphs will be neutral; and (ii) dispersal will be nonlinear. Nevertheless, the linearised equations will take the same form as in the present study. We have studied numerically and analytically invasive waves for two competing morphs in a spatial metapopulation system, and we find the same invasion speeds as in the population model described in the present manuscript.

It is tempting to explain this faster wave speed by saying that mutation between morphs allows the species to exploit the dispersal rate of the disperser and the growth rate of the establisher. Comparing the polymorphic speed 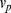 with the speed 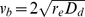 of a `best of both worlds' morph, we find that 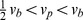 in the parameter range where 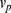 is the invasion speed. The polymorphic wave speed is therefore always slower than the `best of both worlds'. In any case, this explanation is not wholly satisfactory, because it would suggest that the effect would diminish when the mutation rate between morphs becomes small. Since we find a faster polymorphic speed even when the mutation rate is vanishingly small, it appears that the only role of mutation is to ensure that both morphs travel at the same speed, which is one of the assumptions behind the derivation of 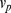. An alternative mechanism for ensuring that both morphs travel at the same speed might lead to the same polymorphic invasion speed even in the complete absense of mutation, though we have not found such a mechanism.

These results suggest that polymorphism is an important factor that needs to be considered when investigating species invasions and so speeds should not be predicted by only looking at the fastest single morph. In terms of the invasion of exotic species this may be of concern if introduced species spread faster than expected threatening native species. However, for species shifting their range as a result of climate change this may be encouraging as it may mean that more species than previously thought will be able to keep up with the rate of change.

Species may experience these faster range expansions as a result of both morphological and behavioural differences in phenotype. For example, many flowering plants exhibit seed polymorphism where large seeds remain near the site of the parent plant and small seeds are wind dispersed to sites further away [28]. These morphological differences may allow faster range expansions to occur. Species may also have different dispersal behaviours, such as the western bluebird *Sialia mexicana*, where aggressive males are more dispersive than non-aggressive males. It has been found that these two phenotypes are maintained in populations as each are advantageous in different stages of range expansions [29].

In insects that have wing polymorphism where one morph is capable of flight and the other is not, trade-offs between dispersal ability and reproductive ability are observed (reviewed in [30]). The anomalous invasion speeds reported in this paper require both traits to differ appreciably – we would expect to see anomalous invasion speeds if the growth rate of the flightless morph is more than twice that of the winged morph, but otherwise invasion would follow the speed of the disperser morph.

We have modelled the invasion here of a species where dispersal ability trades-off with population growth rate. Although in some species, such as the speckled wood butterfly, individuals with increased dispersal ability are less fecund and hence will have slower population growth rates [19], this trade-off is not always observed. It has been suggested that because dispersal and reproductive rate are complex traits it is unlikely that they will directly trade-off against one another and that either may actually trade-off against other traits [31]. For example, Burton *et al.* [12] showed that dispersal and reproduction can trade-off with competitive ability when invading into a landscape occupied by another species. If a competitor is present in the expanding range it may therefore be that the trade-off required for faster invasions may not occur as investing more in competitive ability is more important for a species to be able to expand its range. The relationship between dispersal and reproduction has been found in some species to be positive, with for example, more dispersive cane toads, *Bufo marinus*, having faster growth rates [32] and more dispersive Glanville fritillary butterflies, *Melitaea cinxia*, investing more in reproduction [33], [34]. The result of faster range expansions found using this model may not be transferable to species such as these where there is no trade-off between dispersal and establishment.

A general theory emerging from the literature is that during range expansions there is evolution towards increased dispersal [12]–[15], [35]. There is also a view that spatial sorting can lead to increased dispersal at the range edge, through fast-dispersing individuals dispersing further and then random mating at the range edge of these individuals [36]. While our results agree that having good dispersers at the invasion front allows the population to invade faster, if there is a trade-off in dispersal and establishment ability it is also important to have good establishers present. Indeed under some parameter conditions, for example see Fig. 3C(iii), our results suggest that for faster speeds to occur the density of good establishers at the invasion front is higher than the density of good dispersers. During range expansions, our results suggest that establishment ability (i.e. local population growth rate) is just as important as dispersal ability, and that there will be selection for both to evolve.

These conclusions were made based on the results of a simple deterministic model in which the landscape was modelled as one-dimensional and continuous. These simplifying assumptions were made to make the model analytically tractable but in doing so have made the model less realistic. Modelling using a simple deterministic approach can sometimes give results that are an artefact of the model. Carrying out simulations of a stochastic version of the model will help to determine if these results are robust and will allow us to see when we expect these results to occur. Stochastic simulations may also help to further explain why faster invasions occur when there are differences between the two phenotypes and this will be our next step in this research.

Species expanding their range as a result of climate change are likely to invade into landscapes where the habitat is not continuous and where there may be patches that are unevenly spaced and of different quality [11]. Future work investigating what impact this may have on species invasions may help us to more accurately predict the rate of range expansions as a result of climate change. In this case a more complicated two-dimensional landscape would need to be considered, because a two-dimensional heterogeneous landscape cannot be adequately described by a one-dmensional model. Explicitly modelling a shifting climate would also help us to understand the effect that the loss of suitable habitat at the rear of a species range has on invasions.

The structure of the landscape in terms of availability of habitat and its spatial distribution is also an important factor that needs to be considered for species expanding their ranges. The evolution of dispersal distances and dispersal polymorphism have been found to be affected by landscape structure [37]. It has been predicted that as a result of species having dispersal polymorphism there may be geographic variation in range expansion speeds [37]. Our results implicating that dispersal polymorphism can lead to faster range expansions may lead to even further geographic differences.

In this model we assume perfect heritability of the dispersal trait, however, there is increasing evidence that species responses to climate change can be plastic (reviewed in [38]). Indeed it has been shown that spatial and temporal variation in the environment can result in selection of different dispersal strategies as a result of phenotypic plasticity [39]. If plasticity could result in differences in morphs establishment and dispersal abilities then this could aid the range expansion of species that are not genetically polymorphic. This could result in increased invasion speeds for more species than would be predicted by the present model.

We have shown that the presence of two phenotypes can lead to unexpected results for the speed of biological invasions. Not only can invasion speeds adapt due to evolutionary selection of more invasive phenotypes, but polymorphism itself plays a role in determining invasion speeds. We hope that our results motivate further research to understand the importance of dispersal polymorphism in determining shifting species ranges.

## Supporting Information

Appendix S1 **Detailed derivation of the model from explicit demographic processes.**
(PDF)
