# Supplementary material for: Dispersal Polymorphism and the Speed of Biological Invasions
Source: PLoS One. 2012 Jul 20;7(7):e40496. doi: 10.1371/journal.pone.0040496 (PMC3401290; doi:10.1371/journal.pone.0040496)
Supplement: Appendix S1 — Detailed derivation of the model from explicit demographic processes. (PDF) [file pone.0040496.s001.pdf]

Appendix S1: Model Derivation

Here, we give an example of a set of explicit demographic processes that would lead to the reaction diffusion system (1–2) in the main body of the paper. We first consider a non-spatial two-morph Lotka-Volterra competition model. Let us denote each morph's population density by n_i with with $i \in \{e, d\}$. In this model we assume that density dependence acts on mortality and not on birth. In addition, we assume that there is mutation at birth so that a fraction of the offspring of an individual of morph e is of morph d, and vice-versa. We consider the following demographic rates:

- per capita death rate of morph i is $d_i^0 + d_{i,e}^1 n_e + d_{i,d}^1 n_d$
- per capita birth rate of morph i is b_i
- fraction of morph i births that are of morph $j(\neq i)$ is ν_i

The net growth rate of morph $i(\neq j)$, taking account of birth, death, and mutation, is thus

$$b_i(1 - \nu_i)n_i + b_j \nu_j n_j - n_i(d_i^0 + d_{i,e}^1 n_e + d_{i,d}^1 n_d),$$

which if we then define

$$\begin{aligned} r_i &= b_i - d_i^0 \\ m_{i,j} &= \frac{d_{i,j}^1}{b_i - d_i^0} \\ \mu_i &= b_i \nu_i \end{aligned}$$

leads to the same growth rate term $r_i n_i(1 - m_{i,e}n_e - m_{i,d}n_d) + \mu_j n_j - \mu_i n_i$ as in Eqns. (1) and (2). Note that the mutation rate μ_i is the product of the per capita birth rate and the mutation probability per generation. In order to derive the spatially explicit equations used in the main text, we need (i) to assume that the birth process and density dependence act locally, in which case the local growth rate takes the same form as in the nonspatial case; and (ii) individuals move by small random steps. Taking the diffusion limit for dispersal in the usual way [1] leads to the reaction-diffusion system (1) and (2).

References

[1] Murray J (1993) Mathematical Biology. Berlin: Springer-Verlag, 2nd edition.
